# Miscellaneous Notices

**Published:** 1844-03

**Authors:** 


### Miscellaneous Notices.

*Cheap Dentist*—Is a term, synonymous with *poor dentist,* or rather, *no* dentist, and is regarded by those who know best, as in most instances, designating one of a class of practitioners, who, without a knowledge of even the first rudiments of the science of dental surgery, attempt to practice it; and for the purpose of securing patients, promise to perform all operations pertaining to the art, in the *most skilful* and *scientific manner,* and at *prices so low,* that all, who are not afraid of being duped, may avail themselves of them. If there are any who are disposed to question the correctness of this definition, its truth can be fully attested by most of those who have had the misfortune to fall into the hands of dentists who advertise to perform operations at prices two-thirds less than is usually charged. The operations performed by this description of practitioners, are usually, indeed, cheap, for in most instances, they are not only worth nothing at all, but are oftentimes productive of actual, and not unfrequently, serious, and irreparable injury. And yet, these dentists are patronised. Some go to them because they are not able to pay for such operations upon their teeth as would be really serviceable to them; others are influenced by penuriousness—a false system of economy—and it oftentimes happens, that they pay twice as much in the end, as it would have cost them, to have secured, in the first instance, the services of a competent, skilful practitioner. The first are to be pitied, but the second are deserving of no sympathy. Those who allow themselves to be gulled by every one who calls himself a dentist and advertises cheap operations, have no one to blame but themselves: others again, go to them, not knowing that any special qualification is necessary to practice this branch of surgery, and under the impression that one dentist is as good as another.

The civilized world abounds, at present, with *cheap,* or rather with *quack* dentists. They are to be found in every city, and almost every town in the land, and they will continue to multiply as long as so many seek for *cheap,* rather than *skilful* dentists, and the laws of our country institute no guards around the profession, for the suppression of empiricism.

The tracks of these vandals are daily met with in the mouths of those who have fallen a prey to their ruthless depredations. The injuries they inflict are oftentimes of a very serious nature, and not unfrequently continue through life. They seem to have but one object in view, and that is, to fleece the pockets of their ignorant and credulous dupes. As for a scientific knowledge of the profession they attempt to practice, but which they disgrace, that is a thing with which they would not, on any account, burden their minds, since it is not at all necessary to their success. They can get along quite as well without it as with it, for as for benefitting their patients, that seems the last thing that enters into their calculation.

In our remarks upon cheap dentists, we only wish to be understood as meaning *quack dentists,* who, among the means which they resort to, to gull the unsuspecting, advertise *cheap operations.* We do not recollect to have ever known a scientific, thoroughly qualified and skilful practitioner, to resort to such contemptible trickery. It is beneath the dignity of honorable, high-minded men, and we believe that no one, worthy of confidence, would do it, and in the majority of cases where it is done, our word for it, those who do it, have yet to learn the first principles of correct practice. But while we disapprove of this species of deception, we do not, by any means, wish to be considered as advocating exorbitant charges for dental operations. Those who charge exorbitant prices for their services, are equally destitute of honesty, and generally found to belong to that class of *quack dentists,* who, while they are but little, if any better qualified to practice the art, are possessed of a far greater amount of assurance, and it is to this, that their success is attributable. The scientific and really well qualified of the profession, are generally men, who, while they are constantly devoting their best energies to perfect themselves in the science, that they may the better be enabled to benefit their patients, they expect in return for their services, from those whose circumstances will permit, a fair compensation, and nothing more.

It would be impossible to estimate the amount of injury—the thousands of teeth that are annually destroyed by *cheap* (quack) dentists. The following, from among the cases that are almost daily falling under our observation, will serve as examples of the results that usually follow their operations.

A lady, accompanied by two daughters, one about nineteen and the other seventeen, called upon us, a few days since, for the purpose of obtaining for them, our professional services.

The teeth of both were constituted very much alike, and had been operated on about two years and a half before, those of the eldest by a skilful dentist, but as the mother thought the fee which he charged for his services, a very exorbitant one, she took her other daughter to another dentist, who advertised to perform all operations upon the teeth at one-third the prices usually required for such services. At the time her daughter's teeth were first operated on, the eldest had ten filled and six filed, for which, the dentist charged twenty-five dollars; the youngest had six filled and four filed, and for which, a charge of only eight dollars was made. The mother congratulated herself on having saved, as she supposed, seventeen dollars. Three months, however, had hardly elapsed, before it was found that the decay in the youngest daughter's teeth was still going on. The cheap dentist was again visited, and this time he managed to make a bill of nine dollars, and about six months afterward another of twelve. At the expiration of twelve or fifteen months, the disease being found to be still progressing, the confidence of the mother in the skill of the dentist who had operated on them, had become so much shaken that she determined to consult another, who proved to be of the same class as the last, and after having paid this one thirty dollars, she was induced, by the persuasion of some of her friends, to call upon us. She was accompanied by her eldest as well as by her youngest daughter, and after having given us an account of the operations performed upon the teeth of both, desired that we should do for them whatever we might deem necessary, *provided,* we would be *very moderate* in our charge.

The teeth of the first which had been operated on, were, with the exception of one, in a healthy condition. Nine of the fillings were still perfect—the tenth, by a decay which had commenced in another part of the tooth in which it had been placed, and that had extended to it, was so much loosened that it was no longer of any service. Four or five of her other teeth were decaying, but the disease had not yet progressed so far as to prevent their restoration. The other daughter had lost the crowns of two of her upper incisors, two bicuspides and three molares. The crowns of the two remaining superior incisors and two bicuspides, a cuspidatus, and three molares, were so much decayed as to render their restoration hopeless.

The teeth of the youngest daughter had been filled with gold as well as those of the eldest, but the fillings had not been as skilfully put in. Five, it is true, were still remaining, but they could all have been placed, by a well instructed hand in one cavity.

Now it is obvious that the exercise of this false economy, had not only called for the expenditure of a much larger amount than would have been required to have secured, in the first instance, the services of a skilful practitioner, but had also resulted in the destruction of nearly half the teeth of the youngest daughter.

The *cheap dentists,* in this, as they do, in almost all other instances, prove, ultimately, to be the *dear* ones. We will give one other example before we close our remarks upon this subject.

Mrs. W——, wishing to procure a double set of artificial teeth, applied to Mr. W——, who advertised to insert them at sixty dollars a set. The teeth were obtained, but they did not fit; they caused great irritation to her mouth, and the upper circle did not antagonize properly with the lower. Besides, they tasted so much of copper, that she was apprehensive that their presence in her mouth might be productive of injury to her health. She went back to her dentist a number of times, and stated all of these objections. He tried to persuade her that they were imaginary, he assured her that the plates on which the teeth were set, were pure gold, and that if she would wear the teeth a few months, the jaws would so adapt themselves to the teeth as to cause them to come together properly and fit easily; but, as she could not do this, she determined to go to another dentist. She visited several, and they all told her that the teeth she had, were worth nothing—that they could not be altered so as to make them answer the purpose, and that such a set as would be really serviceable to her, would cost more than double the amount that she had paid for them. Not willing to pay so much, although abundantly able, for a set of artificial teeth, she went to another *cheap* dentist, and obtained another set, which proved to be no better than the first. She now had two sets, but could wear neither, and although her confidence in the art had by this time become greatly shaken, she was nevertheless resolved to make trial of one more set. She had had enough of *cheap* dentists, but still, was anxious to procure a set at as low a price as possible, and for this purpose called upon several dentists—ourself among the number, but as we were so much occupied as to be unable, just at that time, to undertake the operation, she called upon, and obtained the services of a professional friend of ours, who put up for her, a very beautiful and substantial set, for which he charged one hundred and fifty dollars. These teeth she has now worn nearly two years, and having accidentally met with her a few days since, she informed us that she had already been more than compensated, in comfort, for the amount which her last set of teeth had cost her.

In conclusion, we could enumerate, were it necessary, scores of examples as striking as the foregoing, of the bad effects and inutility of the operations of most of those who advertise themselves as *cheap* dentists. There is something suspicious in the name, and those who do it, seem to rest their claim to confidence and patronage upon it, without any reference to qualification or a knowledge of the art, taking it for granted that this will be inferred, if it is but known that they charge less, than what the profession generally do, for their services. By this shallow artifice, hundreds and thousands are being, almost constantly, deceived.—*Balt. Ed.*

---

*Dr. R. Somerby's Concentrated Blow-Pipe and Furnace.*—The inventor of this most admirable contrivance, has done the profession a great service, by furnishing a machine so highly beneficial and useful as is his Concentrated Blow-Pipe and Furnace. Soldering by the ordinary method, is not only hurtful to the lungs, but also to the eyes, which liability to injury is wholly obviated by this apparatus. And while it not only greatly facilitates this operation, it is in every respect, admirably adapted for melting gold and metal for models. To such of the members of the profession as have many artificial teeth to insert, it is invaluable; we had an opportunity of seeing it in operation a few weeks since in the lecture room of the Baltimore College of Dental Surgery, where it was exhibited by Dr. Wm. H. Goddard of Louisville, Ky. and are therefore prepared to commend it to the attention of the profession as worthy of notice. So much, indeed, were we pleased with it, that we at once ordered one for our own use. The cost may deter many from purchasing it, but we believe that might be saved by it in a single year by a dentist in full practice, and as serviceable as it is to dentists, we should suppose, that it would be equally useful to chemists and mineralogists.

*Balt. Ed.*

---

*To Correspondents.*—We regret that Dr. Thackston's very interesting Essay was received too late for insertion in the present number of the Journal. It shall, however, appear in our next, and we hope he will continue to furnish us with contributions from his able pen.

*Gossip with our Profession: by the Junior Editor.*—Much diversity of opinion exists respecting what is the proper shape and curve for the hook or claw of the key instrument for extracting teeth. Some advocate the claims of a section of a circle, some prefer a section of an ellipsis, &c. We have one, (the *only* one we ever saw,) copied after the eagle's claw, which, with a little greater curvature, we made it to resemble as nearly as we could and adapt it to its shaft. To prevent its slipping sideways, we filed the point so as to make it an edge one-sixteenth of an inch wide, and then divided this edge into two points, the dividing notch being about one-twentieth of an inch deep. We like this hook greatly:—because, 1st, it may be applied to a tooth at any point of its side, so as to *rotate* the tooth (an effect sometimes desirable) at the same time that it is rising from its socket, and turning toward the shaft of the instrument. 2dly. It may be applied without much displacement of the gum, thereby lessening the amount of suffering from the operation. 3dly. It has just enough metal, and in just the right place, to be strong enough without being clumsy. Supposing our hook to be an arc, its chord, measuring from the points to the centre of the hole, is about three-fourths of an inch in length, and its versed sine, measuring to the inside of the hook, about half an inch. . . . . Those who would (and who would not?) prefer having their dental plates and clasps bruised and bent as little as possible in the cutting out, are respectfully advised to *saw* them out. Canfield & Brother, of Baltimore, sell a saw-frame and a dozen saws—enough to last a life-time—for $ 1 75! The same gentlemen also sell a host of other things that dentists need, and at rates so very reasonable, that, were it not for the very civil manner in which you are dealt with, you would be surprised to find you had bought so many of them. . . . . Notwithstanding all that has been said, and written, and published, and circulated, all over christendom, about the deleterious, nay, ruinous effects of the mineral acids when used to clean (whiten) the teeth, there are still men in our profession, to its great disgrace, and in this nineteenth century too, when chemistry has been elevated to one of the most beautiful and fascinating of the exact sciences, men who still use, and have, some of them, grown grey in the use of, mineral or other acids, to clean the teeth of their (God help them!) confiding patients. We have ransacked, in vain, every tongue of which we have any knowledge, for terms sufficiently energetic to express our utter abhorrence and strong condemnation of this most reprehensible practice. . . . . The proposition of C. will be acceptable, provided he does not use too much space in advocating things admitted to be correct, nor describe too minutely that which is familiar to nearly all who read this work, as he has done in the article sent for insertion in the Journal. Caoutchouc (not "*catouche*," Mr. C., though we think yours an improvement,) has been for many years, and is now, by many, and we think ought to be, used for separating the teeth, where they do not *need* to be cut away at all. C's objection is evidently against the too common and very pernicious practice of so filing the teeth that the filed surfaces soon are in contact. . . . . Some of the dental instruments sold at the shops seem better fitted for the use of an amateur gardener than a dentist. Only think of having your salivary calculus removed, "in the most scientific manner," with a great triangular hoe! .... In tying ligatures upon the teeth, to regulate them, the first half of the ordinary knot is with difficulty kept tight until the last and binding half is added. Our knot is this: in tying the *first* part, we put one end of the string *twice*, instead of *once*, around the other, and let the knot be in some part of the ligature that is drawn tight upon a tooth. This will not slip if the ligature be waxed. The *last* part is like either half of the ordinary knot. .... The last professional trick to get business, of which we have heard, is, to couple your name with one very nearly like that of some dentists so eminent that even the street they live in is well known thousands of miles away, advertise yourselves as from the same street, (without giving number,) and when asked whether your associate is such or such a one of that name, reply that it is neither, but one of the same family. Thus, you may *dodge* an answer that would convict you of a gross deception, and *palm a lie* upon the dear gullible public. M.

*Washington, Jan.* 1844.

---

*Obituary.*—It is with sincere regret that we are called upon to announce the painful and melancholy intelligence of the decease of Dr. H. H. HAYDEN, Professor of Dental Physiology and Pathology, in the Baltimore College of Dental Surgery, and President of the American Society of Dental Surgeons. He died on Friday, the 26th of January, in the 75th year of his age. With his decease, has fallen one of the brightest stars in the dental profession; to which, as well as to science, his loss will be deeply felt. Engaged in the practice of dental surgery, for about forty-five years, he devoted himself to its study with the most untiring zeal, and during this period contributed many able and valuable papers to the literature of this department of the curative art. Possessed of an investigating and inquiring mind, he, in the mean, extended his researches to several of the collateral sciences, and in some of which, had become eminently distinguished for his profound and critical knowledge. He is the author of a very able and highly interesting work on Geology, of upwards of four hundred pages, published about twenty years ago. But he is gone, and we are called to mourn his loss—a loss which will be severely felt among a large circle of relatives and friends, as well as to suffering humanity.

His life has been a life of usefulness, and had it been consistent with the will of Providence, we could have wished that it had been prolonged for many years yet to come. But although he is no more an inhabitant of earth, his memory still lives, and will be ever cherished by the members of the profession he so long adorned.

The intelligence of this sudden and melancholy event, reaching us as it has, while from home, and just as the last sheets of the present number of the Journal were going to press, we are prevented from making a more extended notice of it at present, but shall avail ourself of a future occasion to speak more at length upon the subject.—*Balt. Ed.*

